# Bayesian modeling of plant drought resistance pathway

**DOI:** 10.1186/s12870-019-1684-3

**Published:** 2019-03-12

**Authors:** Aditya Lahiri, Priyadharshini S. Venkatasubramani, Aniruddha Datta

**Affiliations:** Department of Electrical and Computer Engineering, Texas A&M University, 188 Bizzell St, College Station, 77843 United States

**Keywords:** Bayesian network, Parameter estimation, WRKY transcription factors, Network inference

## Abstract

**Background:**

Plants are sessile organisms and are unable to relocate to favorable locations under extreme environmental conditions. Hence they have no choice but to acclimate and eventually adapt to the severe conditions to ensure their survival. As traditional methods of bolstering plant defense against stressful conditions come to their biological limit, we require newer methods that can allow us to strengthen plants’ internal defense mechanism. These factors motivated us to look into the genetic networks of plants. The WRKY transcription factors are well known for their role in plant defense against biotic stresses, but recent studies have shed light on their activities against abiotic stresses such as drought. We modeled this network of WRKY transcription factors using Bayesian networks and applied inference algorithm to find the best regulators of drought response. Biologically intervening (activating/inhibiting) these regulators can bolster the defense response of plants against droughts.

**Result:**

We used real world data from the NCBI GEO database and synthetic data generated from dependencies in the Bayesian network to learn the network parameters. These parameters were estimated using both a Bayesian and a frequentist approach. The two sets of parameters were used in a utility-based inference algorithm to determine the best regulator of plant drought response in the WRKY transcription factor network.

**Conclusion:**

Our analysis revealed that activating the transcription factor WRKY18 had the highest likelihood of inducing drought response among all the other elements of the WRKY transcription factor network. Our observation was also supported by biological literature, as WRKY18 is known to regulate drought responsive genes positively. We also found that activating the protein complex WRKY60-60 had the second highest likelihood of inducing drought defense response. Consistent with the existing biological literature, we also found the transcription factor WRKY40 and the protein complex WRKY40-40 to suppress drought response.

**Electronic supplementary material:**

The online version of this article (10.1186/s12870-019-1684-3) contains supplementary material, which is available to authorized users.

## Background

The global population is set to rise by 34% by the year 2050, and increasing crop yields to ensure food security has become a grand challenge [1]. The rise in temperature worldwide due to global warming has increased the risk of droughts affecting crop yields and has further complicated this challenge. Studies have shown that the global drought-affected area will rise significantly by 2050, and it will be accompanied by a sharp drop in crop yield [2]. The unprecedented rise in worldwide population accompanied by a rise in demand for crop supply comes at a time when traditional approaches of maximizing crop production are coming to their biological limits. Hence, developing drought resistant crops has become a global priority to ensure food security. Fortunately, plants have multiple innate stress sensing mechanisms that can detect unfavorable changes in the environment and deploy appropriate defense responses. Therefore, it is of great interest to understand the genetic networks behind a plant’s defense mechanism to augment its genetic yield potential while reducing its susceptibility to harsh conditions.

Abscisic acid (ABA) is a well-known plant hormone that is induced under drought stress conditions and regulates a plant’s gene expression through the action of transcription factors [3, 4]. The family of WRKY transcription factors is traditionally associated with plant defense mechanisms against pathogens; however, many recent studies have highlighted WRKY’s role in abiotic stress responses [5–7]. Since WRKY is one of the largest families of transcription factors in plants with such diverse roles in plant defense mechanisms, it is practical to model the interaction among various components of the WRKY’s signaling pathway to gain valuable insights into these interactions [8]. In this paper, we use Bayesian networks (BN) to model the ABA-induced WRKY transcription factor network. We then apply a utility-based inference technique to determine the significant regulators of drought stress response genes in the BN. This approach allows us to integrate existing biological knowledge into our model.

### *Review of biological background*

Similar to the way adrenaline functions as a stress hormone in animals, plants respond to harsh environmental changes, pathogen attacks or wounding by secreting plant hormones, such as ABA, Cytokinins, Salicylic Acid and Ethylene to trigger its’ own defense mechanisms. In the context of plants, droughts are characterized by the unavailability of water, which can prevent plants from performing basic survival processes such as photosynthesis. When a plant faces water deficit conditions, it can defend itself either by the process of avoidance or tolerance. In the case of avoidance, a plant may complete its life cycle in the wet season. Whereas in the case of tolerance, the plant may initially acclimate to the change in conditions by introducing reversible changes into its physiology through altering its gene expression; however, if drought conditions still persist, then the plant passes its altered genes to its next generation so that these new generations of plants are already adapted to the drought conditions [9].

To adopt either of these defense mechanisms, the plant must undergo a process of signal transduction when it gets the initial cue of droughts,such as a drop in the water potential in the apoplast and a rise in the ion concentration [9]. All these signals along with many others cause a rapid rise in the level of the plant hormone ABA, which acts as a stress sensor, and it subsequently activates secondary messengers, such as *Ca*^2+^, Reactive Oxygen Species (ROS) and Cyclic Adenosine Monophosphate (cAMP). These secondary messengers turn on their respective signaling pathways (e.g., MAPK, CDPK) where protein phosphorylation (addition of phosphate ${PO_{4}^{3-}}$) and dephosphorylation may take place via the actions of kinases (enzymes) and phosphatases (enzymes) respectively [10]. Following the signaling action of kinases and phosphatases, transcription factors are either activated or deactivated to regulate downstream gene expression [10]. Transcription factors are proteins that bind to a specific DNA sequence of the gene(s) to activate or deactivate them. Finally, transcription factors are directly responsible for turning on the stress response genes and turning off any other nonessential genes.

**Fig. 1:**
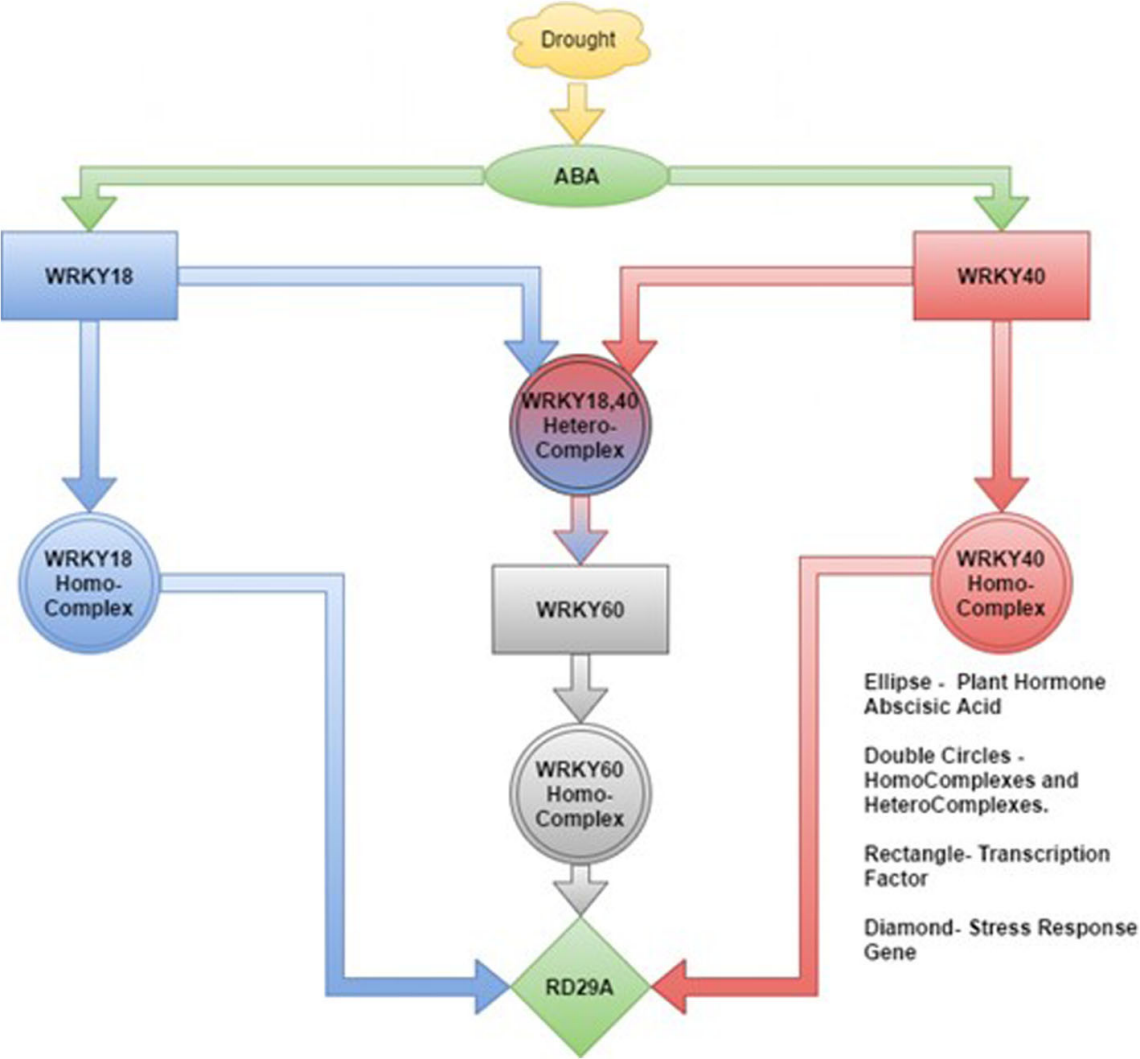
The induction of WRKY transcription factor signaling pathway by ABA. Under drought conditions the plant hormone ABA gets activated. ABA then initiates the activation downstream drought response genes via the actions of transcription factors and protein complexes

Each family of transcription factors, such as WRKY, bZIP, and NAC regulates a large number of genes. Hence, learning the activities of transcription factors is critical for understanding the stress response mechanisms in plants. WRKY is a large family of transcription factors and has roles in plant defense mechanisms against both abiotic and biotic stress. Until recently, the role of WRKY in dealing with abiotic stresses was not as extensively explored as in the case of biotic stresses. Due to these reasons there is a lack of available experimental data [3]. In this paper, we are interested in studying the interactions among various members of the WRKY transcription factor signaling pathway (Fig. 1) which are rapidly induced by ABA under drought stress. Learning these interactions can give us more profound insights into the functioning of this pathway, which can aid us in developing intervention strategies for breeding drought resistant plants.

It has been shown that ABA induces the transcription factors WRKY18, WRKY40, and WRKY60 under water deficit and salt stress conditions (Chen et al.) [11]. Furthermore, it has also been reported that WRKY18 and WRKY60 have positive sensitivity for ABA in inhibition of seed germination, root growth and enhancing plant sensitivity to water deficit stress; in contrast, WRK40 antagonizes WRKY18 and WRKY60 to affect a plant’s ABA sensitivity and abiotic stress responses (Chen et al.). Experiments were carried out with WRKY18 and WRKY40 deficient mutants, which showed that the expression of WRKY60 was negligible. This implied that WRKY18 and WRKY40 directly induced WRKY60 by recognizing a cluster of W-BOX sequences in the promoter of WRKY60 (Chen et al.). In addition to the various regulatory behaviors of these three WRKY transcription factors, it has been noted that these three transcription factors not only interact with themselves to form three homocomplexes but also, interact amongst each other to form heterocomplexes [12].

## Methods

### *Bayesian network modeling*

Biological networks are inherently tortuous and stochastic. It is often difficult to interpret the multivariate interactions among different components of the network. A BN is a directed acyclic graph that determines the conditional decomposition of the joint probability distributions of a set of random variables in the network and thus simplifies the computation of their joint probability distribution (Sinoquet and Mourad) [13]. Therefore, we are interested in using BNs to model the interactions in a biological network as they provide a clean and compact framework for representing the joint probability distributions and for drawing inferences from these networks [14]. Inspection of BNs can help enhance our beliefs about relationships among different elements in the network and provide insights into the causality of the network.

**Fig. 2:**
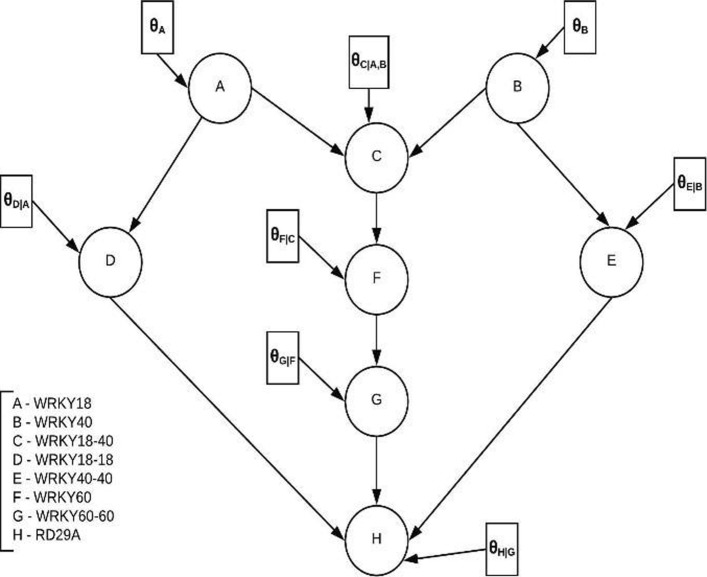
BN model of WRKY signaling pathway with conditional probabilities depicted in rectangles. Every circle in the BN is a binary random variable with states 0 and 1 corresponding to inhibition and activation respectively. The rectangular boxes represent the probability with which each node gets activated. The arrows represent causal biological relationship between nodes

In this paper, we model the WRKY signaling pathway (Fig. 1) involved in the drought stress responses of the model plant, Arabidopsis. Based on the signal transduction pathway outlined in Fig. 1, we have constructed a BN as shown Fig. 2. Each circular node (A,B,C, …,H) represents a gene, transcription factor or protein complex, and every directed edge between the nodes represents a causal relationship that exists in the WRKY signaling pathway. Attached to every node is a rectangle, which represents the parameter or local probability model associated with that node. For instance, *θ*_*C*|*A*,*B*_ represents the conditional probability density of node C given its parent nodes A and B. These parameters can be learned from data and are important in the understanding the overall graph structure.

### *Parameter estimation*

Depending on the availability of prior knowledge in a given application, one may use a frequentist approach or Bayesian approach for learning the parameters of a BN. Frequentist approaches, such as Maximum Likelihood Estimation (MLE) assume that the parameter being learned is fixed and produces a point estimate without taking into account prior information. On the other hand, Bayesian Estimation treats the parameter as a random variable and uses the data and prior distribution of the parameter to obtain the parameter’s posterior distribution. Furthermore, the Bayesian approach takes into account the problem of zero probability estimates, which may affect the learning algorithm. Bayesian methods provide a non-zero probability estimate even when the prior information follows a uniform distribution (non-informative prior). This is because the posterior belief is being governed both by data, and prior knowledge, and hence zero estimates of probability are only associated with non-occurrence of an event. However, the Bayesian estimation process is computationally challenging as it requires performing integration in order to obtain the probability of the evidence (data). Due to this reason, we use the concept of conjugate priors for a given likelihood function in the process of Bayesian estimation [15].

In the BN in Fig. 2, we assume that each of the nodes X in the BN can attain only binary values, X = 0 or X = 1. When X =1 for a node, it indicates that the gene, transcription factor or protein complex represented by that node is activated, whereas if X =0, it indicates just the opposite (gene, transcription factor or protein complex is inhibited). This formulation allows us to model the state of each node in the network, given the state of its parent nodes, using a Bernoulli distribution. Now, consider a BN with N nodes such that *θ*_*X*_ be the probability that X =1 (success) and 1- *θ*_*X*_ be the probability that X =0 (failure). Assume that we make n (>0) observations regarding the state of each node and we let k(≤ n) be the number of times the state of a node is 1. We further assume that the sequence of random variables X1, X2, …,Xn obtained after n observations for each node to be independent and identically distributed. So, the probability distribution of a node given its parent nodes (*P*_*a*_(*X*)) follows a Binomial distribution and is given by:

1 $$  P(X|P_{a}(X),\theta_{X}) \sim Binomial(n,\theta_{X})  $$

2 $$  Binomial(n,\theta_{X})=\frac{(n)!}{(n-k)!k!} \theta^{k}_{X} (1-\theta_{X})^{n-k}  $$

To estimate the posterior distribution, we need to define the prior over the parameter *θ*_*X*_ for our model. Since the likelihood function associated with our model is binomial, we choose the prior distribution to follow a Beta distribution with some shape parameters (*α*_*X*_, *β*_*X*_), and this results in the representation:

3 $$  \theta_{X} \sim Beta(\alpha_{X},\beta_{X})  $$

Due to the modeling of the priors as a beta distribution under Binomial likelihood, it follows from the properties of conjugate families that the posteriors will also follow a beta distribution with shape parameter $\left (\alpha ^{'}_{X},\beta ^{'}_{X}\right)$ [15]. In our model, the posterior distribution of the parameter *θ*_*X*_ is given by:

4 $$  P(\theta_{X}|X) \sim Beta(\alpha^{'}_{X},\beta^{'}_{X})  $$

where $\alpha ^{'}_{X} =$ (*α*_*X*_ + k) and,$\beta ^{'}_{X} =$ (*β*_*X*_ + n – k). The expected value of this distribution is given by:

5 $$  E\left[\theta_{X}|X\right]=\frac{\alpha^{'}_{X}} {\left(\alpha^{'}_{X}+\beta^{'}_{X}\right)}  $$

We can use experimental data to iteratively update *α*_*X*_ and *β*_*X*_ to obtain the posterior distribution. With more data, the posterior distribution will converge towards the actual posterior distribution. We modeled the prior as a Beta under Binomial likelihood and, this allowed us to obtain a closed form solution for the posterior. Other non-conjugate priors may be used; but, a closed form solution may not be guaranteed. Note that this approach gives us the marginal and conditional posterior distribution associated with every node and not their probabilities (*θ*_*X*_, *θ*_*Y*|*X*_). In this paper for the purpose of learning these probabilities, we approximate the probabilities by the expected value (Eq. 5) of the posterior distribution for their respective nodes. Furthermore, we also learn the probabilities using the frequentist approach of MLE, in order to compare the final results, we get by using both the approaches. Ideally, when data is abundant, the Bayesian approach and MLE estimate converge to the same point [16]. The marginal probabilities and the conditional probabilities for binary random variables can be estimated using MLE by Eqs. 6 and 7 respectively.

6 $$  \theta_{X_{1}}=\frac{M\left[X^{1}\right]}{M\left[X^{1}\right]+M\left[X^{0}\right]}  $$

7 $$  \theta_{Y_{1}|X_{0}}=\frac{M\left[Y^{1},X^{0}\right]}{M\left[Y^{1},X^{0}\right]+M\left[Y^{0},X^{0}\right]}  $$

Where M[ *X*^1^] is the number of times the random variable X is 1, M[ *X*^0^] is the number of time X is 0, M [ *Y*^1^, *X*^0^] is the number of times X is 0 and Y is 1 and M[ *Y*^0^, *X*^0^] is the number of times X is 0 and Y is 0. A key assumption we make in the BN modeling is that the joint distribution for the set of nodes factorizes according to the BN in Fig. 2. This assumption implies that dependencies in the biological structure are reflected in the data from which we are learning the network parameters. One can employ constraint-based or score-based learning techniques to derive the graph structure from data, and then subsequently learn the network parameters [17]. However, in the context of this paper, we avoid learning the graph structure as publicly available experimental data is highly limited for the WRKY transcription factor under abiotic stress, and also our network contains protein complexes (nodes C, D, E, and G) for which expression data doesn’t exist alongside with gene expression data (nodes A, B, F, and H). Generally, datasets that contain gene expression data do not contain expression data for protein complexes, and vice versa. Hence synthetic data were generated for the protein complexes using the dependencies in the BN and the experimental data for other non-protein complex nodes in the network for which data were available.

### *Utility based inference in Bayesian networks*

After the network parameters or the local probabilities associated with every node are inferred from the data, the BN has sufficient information for carrying out inference. Our objective is to find a single node in the WRKY BN that maximizes the upregulation of the downstream expression of the drought resistant gene. In other words, we are interested in finding a single node (nodes A-G) in BN, which when up or downregulated maximizes the chances of our stress response gene (node H) being upregulated.

**Fig. 3:**
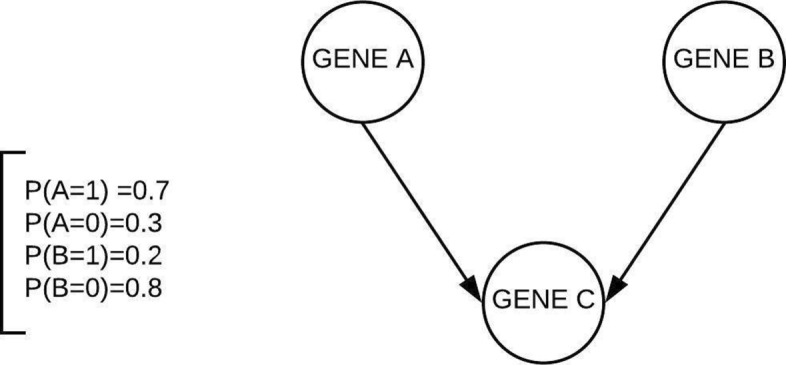
Example BN, with marginal probabilities of parent nodes. This BN depicts the causal and probabilistic interactions that exist among genes A, B and C

There are multiple ways to perform inference in a BN. Pearl’s message passing algorithm is favored whenever we have a singly connected graph as it allows us to perform exact inference [18]. However, the BN in consideration here is not singly connected and also has loops, which cannot be handled using Pearl’s algorithm. Other non-exact sampling-based techniques require a large amount of data to provide reliable inference. Hence in this paper, we have considered another type of approximate inference technique that computes a score, commonly known as expected utility, based on an action taken at a specific node. Utility measures the efficacy of that action. To implement utilities into our BN, we first need to understand the concept of Bayesian decision networks, and how we can create one from a BN. In order to illustrate these concepts, consider the following example involving a simple BN as shown in Fig. 3.

**Fig. 4:**
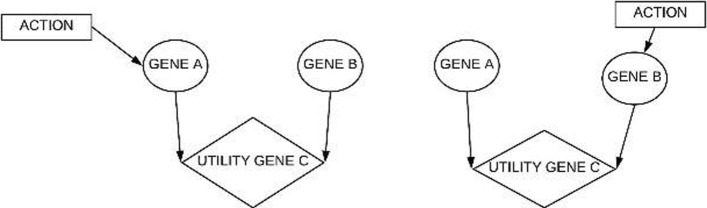
Bayesian Decision networks for intervention at gene A and at gene B. The rectangular box represent the action node.The circular nodes represent random variables and the diamond shaped node denote utility nodes

The BN in Fig. 3 has three nodes gene A, gene B, and gene C and we assume each can take on a binary value of 0 (inhibited) or 1 (activated). Gene A and gene B are parent nodes of gene C, and have marginal probabilities associated with them as shown in Fig. 3. Also, let us assume that when gene A is active it activates gene C and when gene B is active it inhibits gene C. Based on this BN we construct a Bayesian decision network as shown in Fig. 4. The rectangular node acts as a decision (action) node, the diamond-shaped node serves as a utility node and the circular nodes represent chance (nature or probabilistic) nodes. In this example we are interested in having gene C to take on the value of 1, this is what the expected utility will measure. In this case we have the option to take action at either of the chance nodes gene A or gene B. Once we decide to take action at a chance node, it no longer remains a chance node but becomes a deterministic node.

Depending on the action taken the expected utility can be calculated by Eq. 8:

8 $$ EU(A)=\sum\limits_{i}P(O_{i}|A) U(O_{i})  $$

where P(*O*_*i*_|*A*) represents the probabilities of the outcomes (*O*_*i*_) that are consistent with action A, and U(*O*_*i*_) represents the utility value for that outcome under action A. The utility table is defined in Table 1, where the first row represents the best-case scenario when gene A is active and gene B is inhibited, and the last row represents the worst-case scenario when gene A is inhibited and gene B is active. The rest of the rows represent the other possible scenarios. The utility scores assigned are relative to best (highest utility) and worst (lowest utility) case scenarios, these values are not unique and can be redefined differently, however, the scores must reflect the scenario depicted in the decision network.

**Table 1:** Utilities for example Bayesian decision network

Gene A	Gene B	Utility Gene C
1	0	100
1	1	50
0	0	50
0	1	0

Using Eq. 8 we first calculate the expected utility for taking action at gene A as follows:

Case 1: Action taken: gene A is activated (A = 1).

$$\begin{array}{*{20}l} &EU(A=1)=P(A=1,B=1|A=1)\\ &*U(A=1,B=1)+P(A=1,B=0)\\ &*U(A=1,B=0)\\ &=P(B=1)*U(A=1,B=1)+P(B=0)\\ &*U(A=1,B=0)\\ &=0.2*50 + 0.8*100= 90\\ \end{array} $$

Case 2: Action taken: gene A is inhibited (A = 0).

$$\begin{array}{*{20}l} &EU(A=0)=P(A=0,B=1|A=0)\\ &*U(A=0,B=1)+P(A=0,B=0|A=0)\\ &*U(A=0,B=0)\\ &=P(B=1)*U(A=0,B=1)+P(B=0)\\ &*U(A=0,B=0)\\ &=0.2*0 + 0.8*50=40\\ \end{array} $$

So, when gene A =1 or activated the expected utility is greater. Similarly, let us calculate the expected utilities for taking action at gene B.

Case 1: Action taken: gene B is activated (B = 1).

$$\begin{array}{*{20}l} &EU(B=1)=P(A=1,B=1|B=1)\\ &*U(A=1,B=1)+P(A=0,B=1|B=1)\\ &*U(A=0,B=1)\\ &=P(A=1)*U(A=1,B=1)+P(A=0)\\ &*U(A=0,B=1)\\ &=0.7*50 + 0.3*0=35\\ \end{array} $$

Case 2: Action taken: gene B is inhibited (B = 0).

$$\begin{array}{*{20}l} &EU(B=0)=P(A=1,B=0|B=0)\\ &*U(A=1,B=0)+P(A=0,B=0|B=0)\\ &*U(A=0,B=0)\\ &=P(A=1)*U(A=1, B=0)+P(A=0)\\ &*U(A=0,B=0)\\ &=0.7 *100 + 0.3*50 = 85\\ \end{array} $$

Hence when gene B is inhibited the expected utility is larger. However, the utility of gene A being activated is larger than gene B being inhibited. So, we must select activating gene A over inhibiting gene B to maximize the chances of gene C being activated.

## Datasets and simulations

To estimate the parameters and carry out utility calculation in the BN, we need to obtain data for WRKY transcription factor under drought stress condition. Since the WRKY transcription factor has only recently been implicated for its role in abiotic stress response, it is difficult to obtain large scale data that is publicly available. However, we were able to obtain real world microarray gene expression data for all the genes and transcription factors (Nodes A, B, F, and H) in the BN from the datasets GSE46365, GSE65046, and GSE76827, which are publicly available from the NCBI GEO database [19–21]. These datasets were individually normalized and binarized and aggregated into one composite dataset, which contained 116 data points for each of the non-protein complex nodes (genes and transcription factors). Once the real-world data were binarized, they were used along with the dependencies in the BN to generate data for the protein complexes denoted by nodes C, D, E, and G. For example, in order to generate the dataset for node D the expression values for node A and node H were observed, i.e. all the parents and children of node D. If both node A and H were observed to be upregulated (state =1) node D was assigned deterministically to be upregulated (state =1), if both the nodes A and H were both observed to be downregulated (state =0) then node D was assigned deterministically to be downregulated (state =0). This is because we know from the biological literature (Chen et. al) that node A upregulates node D, and node D, in turn, upregulates Node H. If the expression status of node A was upregulated (state =1) and that of node H was downregulated (state =0) then node D was assigned a value of 1 (upregulated) with a probability larger than 0.5. This is because if node A is upregulated it is highly likely that node D is also upregulated but not enough to counter the downregulatory effect of node E on node H, which might have caused node H to be downregulated. The probability with which node D was upregulated was randomly selected from a set of discrete probability values of [0.6,0.7,0.8 0.9 and 1] where each value had an equally likely chance of being selected. Similarly, when node A =0 and node H =1 node D was probabilistically assigned a value of 0 (downregulated). In this fashion the data for nodes C, G, and E were also generated, so that these synthetic data reflected the network dependencies and the real data for the nonprotein complex nodes. Tables 2, 3, 4, 5, Pseudocode 1 given below along with the R code attached in the additional files section (Additional files 1, 2, 3, 4, 5, 6, 7, 8, 9 and 10) further explain the data generation for nodes D, E, G and C. Generally, gene expression datasets do not contain protein-protein interaction data, which is needed for avoiding generating synthetic data for protein complexes in our network. Though we can find protein-protein interaction datasets, however, those datasets will not contain gene expression data, so in order to circumvent this issue, we considered generating synthetic data for the protein complexes in our BN.

**Table 2:** Synthetic Data generation for Node D

Node A	Node H	Node D
1	1	1
1	0	Assign(Value=1)
0	1	Assign(Value=0)
0	0	0

**Table 3:** Synthetic Data generation for Node E

Node B	Node H	Node E
1	1	Assign(Value=1)
1	0	1
0	1	0
0	0	Assign(Value=0)

**Table 4:** Synthetic Data generation for Node G

Node F	Node H	Node G
1	1	1
1	0	Assign(Value=1)
0	1	Assign(Value=0)
0	0	0

**Table 5:** Synthetic Data generation for Node C

Node A	Node B	Node F	Node C
0	0	0	0
0	0	1	Assign(Value =0)
0	1	0	Assign(Value =0)
0	1	1	Assign(Value =1)
1	0	0	Assign(Value =0)
1	1	0	Assign(Value =1)
1	0	1	Assign(Value =1)
1	1	1	1

Once synthetic data for all the protein complex nodes were generated, they were aggregated along with real world data in a single dataset. This dataset was used for the purpose of estimating the network parameters using the Bayesian approach and the maximum likelihood approach as outlined in section on parameter estimation. For the Bayesian approach, the prior for every node was first initialized to a beta (1,1) distribution, which is a uniform distribution over the interval [0,1]. Using the data and Eq. 4 the posterior distribution for every node was updated and the expected values were computed using Eq. 5. The expected values were approximated to be the conditional probability for the nodes. A separate set of parameters were learned using the MLE approach as well using Eqs. 6 and 7.

Then, the utilities for single point intervention were computed. The utility node was set at node H, and the utility analysis was carried out to find single intervention points that upregulated node H. A utility table (Table 6) was defined based on the Bayesian decision network. The utility at node H depended directly on nodes D, G and E. The best case scenario was when nodes D and G were upregulated and node E was downregulated, whereas the worst case scenario was when nodes D and G were downregulated and node E was upregulated. These scenarios were representative of the actual biological processes in the network, and the utility scores were defined relative to these scenarios, with a high utility score being favorable. Simulations were carried out using R software [22], and the utility calculations were done using Netica [23].

**Table 6:** Utility values used to calculate the maximum expected utilities in Figs. 6 and 7

Node G	Node D	Node E	Utility at Node H
1	1	1	50
1	1	0	100
1	0	1	10
1	0	0	50
0	1	1	10
0	1	0	50
0	0	1	0
0	0	0	10


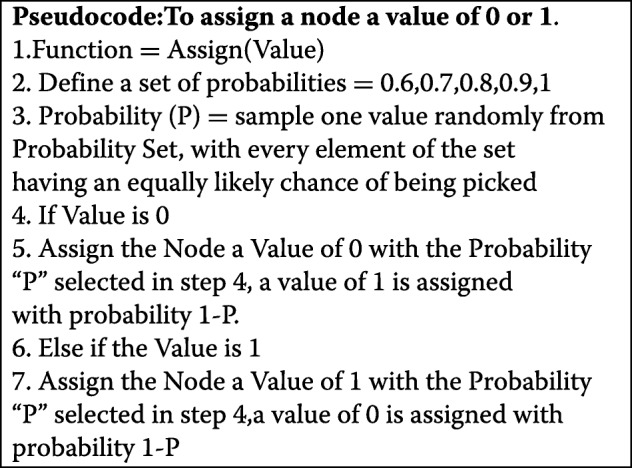


## Results

**Fig. 5:**
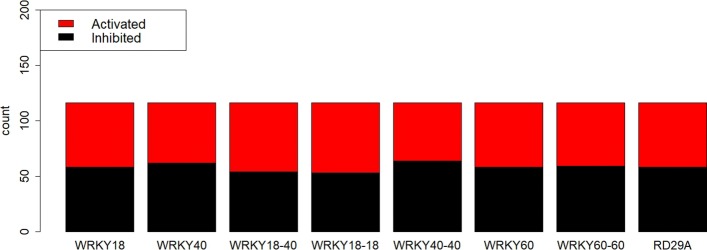
Node activation vs inhibition plot. The red region in the bar represents the instances a particular node was activated whereas the black region in the bar represents the instances when that node was inhibited

**Fig. 6:**
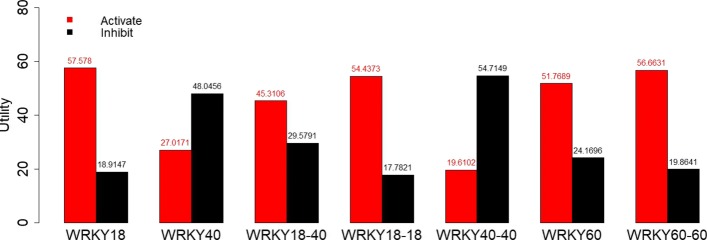
Maximum expected utility values when using parameters from the Bayesian approach. The red bars represent the utility score for activating a node and the black bars represent the utility score for inhibiting that node. Intervening at the node with the highest utility score offers the best chance for upregulating downstream drought response genes

**Fig. 7:**
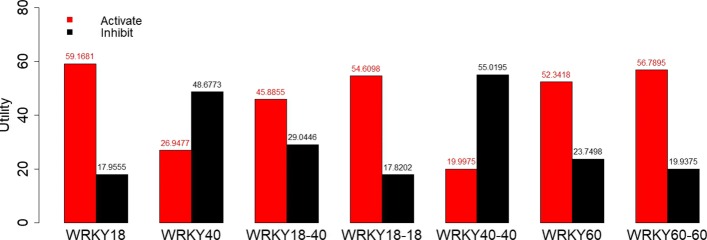
Maximum expected utility values when parameters are estimated using MLE. The red bars represent the utility score for activating a node and the black bars represent the utility score for inhibiting that node. Intervening at the node with the highest utility score offers the best chance for upregulating downstream drought response genes

The divided bar plot in Fig. 5 represents the activation and inhibition status for every gene in the BN, after the data have been preprocessed. Table 7 displays the conditional probabilities estimated using the Bayesian and MLE approaches. The maximum expected utilities using the parameters obtained from Bayesian and MLE approach are displayed in Figs. 6 and 7 respectively.

**Table 7:** Marginal and Conditional Probabilities learned using the Bayesian and MLE approaches

Local Probabilities	Bayesian Approach	MLE Approach
P(A1)	0.50	0.50
P(B1)	0.466	0.465
P(C1 |A1,B1)	0.905	0.925
P(C1 |A0,B1)	0.625	0.645
P(C1 |A1,B0)	0.60	0.611
P(C1 |A0,B0)	0.13	0.113
P(D1 |A1)	0.983	1
P(D1 |A0)	0.10	0.086
P(E1 |B1)	0.857	0.870
P(E1 |B0)	0.093	0.080
P(F1 |C1)	0.766	0.774
P(F1 |C0)	0.196	0.185
P(G1 |F1)	0.867	0.879
P(G1 |F0)	0.117	0.103

From the utility analysis using both the Bayesian and MLE approaches, we find that WRKY18 has the highest utility score for its activation. This means that the upregulation of WRKY18 is the most effective single-gene intervention in bringing about the upregulation of the downstream drought stress response gene. This result is consistent with the biological literature, which suggests that WRKY18 has a positive sensitivity to ABA under drought stress conditions and plays a critical role in the upregulation downstream gene expression. We can also see from the bar plots in Figs. 6 and 7 that the second-best point for intervention is at the protein complex WRKY60-60, which also upregulates the expression of the downstream drought stress response genes. Also, consistent with the literature we see that both WRKY40 and WRKY40-40 have high utilities for inhibition, as they are responsible for downregulating the downstream drought stress response gene. We also see that our utility scores in both the Bayesian and MLE approaches are comparable, which is due to the fact that the estimated probabilities (Table 7) using both the approaches are very similar.

## Discussion

In this paper, we presented the WRKY signaling pathway, which is traditionally associated with plant defense response against biotic stresses, but recently it has been shown to play a significant role in plant defense response against abiotic stresses, such as droughts. Due to its diverse role in plant defense, it was an interesting pathway choice to investigate. We modeled the WRKY pathway using a BN, where every node in the network represented a gene, transcription factor or protein complex from the pathway and every edge between the nodes represents a causal relationship that exists in the pathway. Associated with each node is a conditional or marginal probability, which represents the probability with which the node is activated or inhibited. For our analysis, we assume that nodes in the network can only take binary values of 1 (activation) or 0 (inhibition). Since a BN can capture both the causal biological relationships and the probabilistic nature of biological pathways, it was an ideal choice for modeling purposes.

In order to learn the parameters in the network, we used real-world gene expression data and generated synthetic data, which reflected the network dependencies. To estimate the local conditional and marginal probabilities both a Bayesian approach and a frequentist approach were used. In the Bayesian approach, we assumed every node to have a prior distribution of Beta (1,1) (uniform distribution in the range [0,1]), which signified we had no prior knowledge about our model. Since our likelihood followed a binomial distribution, we were able to use a closed form formula through the properties of conjugate families to arrive at a posterior distribution for each node. The expected value of the posterior distribution was used as an estimate for the local probabilities. We selected conjugate families in order to simplify our calculations, and arrive at a closed form solution, however, it may not always be the best choice to select a conjugate prior. If sufficient information is available, the prior can be modeled using non-conjugate family distributions, and the posterior can be estimated using (Markov-Chain-Monte-Carlo) MCMC techniques, although, this may be computationally expensive. In the frequentist approach, we simply employed the maximum likelihood estimate to obtain the local probabilities. The probabilities obtained using both the methods were found to be very similar to each other.

Once the parameters from each method were learned, the task of inferring the best node for intervention was carried out using the concept of utilities. We used a non-exact inference technique in our model as we could not employ exact techniques, such as Pearl’s message passing algorithm in our Bayesian network as the former works only for singly connected, and loop less networks. Also, the number of data points was quite limited, which made the choice of utility for the purpose of inference quite sensible as opposed to data intensive sampling-based inference techniques. Furthermore, utility based inference can be easily applied to larger BN. The utility analysis carried out using parameters from the Bayesian and MLE approaches revealed that WRKY18 served as the most potent node for intervention when upregulated. Therefore, upregulating WRKY18 would further upregulate the downstream stress response genes in the WRKY signaling pathway. This result was consistent with the biological literature, which says that WRKY18 actively upregulates the gene expression of drought response genes under drought conditions. Our next step in this research will be to explore and implement more informative priors in the Bayesian parameter estimation approach rather than using non-informative Beta (1,1) prior that we have used here since our study was limited by the lack of prior knowledge regarding the network. We would also like to investigate other signaling pathways that are implicated in plant defense response against droughts, and find the key regulators in those networks, and compare their efficacy to that of WRKY18. We limited the scope of our research to only single node intervention, in the future we would also like to expand our research to multiple node interventions and study how these interventions regulate multiple downstream genes.

## Conclusion

We modeled a drought responsive singling pathway in a plant using Bayesian networks, and applied a utility-based inference algorithm, which revealed that WRKY18 upon its activation had the best chance of activating downstream drought resistance gene. This result was found to be consistent with the biological literature along with the rest of the results from the utility-based analysis. In the future, we plan to employ CRISPR-CAS9 to activate WRKY18 in plants in the field to measure the efficacy of WRKY18 in fighting against droughts. This process of using Bayesian networks to find regulators of drought response can be applied to find key regulators in other plant networks, which can be useful for creating robust and valuable crops in the future.

## Additional files

Additional file 1 R code for generating synthetic data and estimating parameters. Also serves as the main execution file. (R 5.02 kb)

Additional file 2 R code for plotting utilities. (R 1.12 kb)

Additional file 3 R code for finding MLE. (R 1.06 kb)

Additional file 4 R code to normalize the data. (R 1 kb)

Additional file 5 R code to calculate shape parameters of Beta distribution. (R 4.20 kb)

Additional file 6 R code to binarize the data using mean or median. (R 1 kb)

Additional file 7 R code to generate synthetic data. (R 1 kb)

Additional file 8 Excel file containing the data used from dataset GSE46365 to support the conclusion of this article.The complete dataset can be publicly accessed online from NCBI GEO database with the accession number of GSE46365. (XLSX 9.89 kb)

Additional file 9 Excel file containing the data used from dataset GSE65046 to support the conclusion of this article.The complete dataset can be publicly accessed online from NCBI GEO database with the accession number of GSE65046. (XLSX 10.1 kb)

Additional file 10 Excel file containing the data used from dataset GSE76827 to support the conclusion of this article.The complete dataset can be publicly accessed online from NCBI GEO database with the accession number of GSE76827. (XLSX 9.03 kb)

## Abbreviations

ABA: Abscisic acid
BN: Bayesian network
ROS: Reactive oxygen species
cAMP: Cyclic adenosine monophosphate
MLE: Maximum likelihood estimation
MEU: Maximum expected utility
MCMC: Markov chain Monte Carlo
